# Psoriatic arthritis patients have increased morbidity already at the time of diagnosis: a case–control study

**DOI:** 10.1007/s00296-026-06212-2

**Published:** 2026-07-06

**Authors:** Anna Laine, Paula Muilu, Hannu Kautiainen, Vappu Rantalaiho

**Affiliations:** 1 Faculty of Medicine and Health Technology, Tampere University, Tampere, Finland; 2 Department of Medicine, Kanta-Häme Central Hospital, Sairaalakatu 3, 13530 Hämeenlinna, Finland; 3 Centre for Rheumatic Diseases, Tampere University Hospital, Tampere, Finland; 4 Primary Health Care Unit, Kuopio University Hospital, Kuopio, Finland; 5 Folkhälsan Research Center, Helsinki, Finland

**Keywords:** Arthritis, Psoriatic, Comorbidity, Prevalence, Epidemiology

## Abstract

To evaluate the prevalence of comorbidities preceding the diagnosis of psoriatic arthritis (PsA) compared with the general population. We identified all adult patients granted a special reimbursement (SR) for disease-modifying anti-rheumatic drugs for PsA between 2002 and 2014 from the Finnish Social Insurance Institution register (*N* = 6077). Three general population controls were matched to each case. Visits to specialized medical care (Classification of Disease 10th revision coded), and prescription drug purchases categorized by Anatomical Therapeutic Chemical codes were collected for cases and controls between 2000 and 2014. We evaluated the period from 24 months before to 3 months after the index date (ID), defined as the date when the SR was granted. SRs granted for other chronic diseases prior to the ID were additionally collected. PsA patients had three times more visits to specialized medical care and increased overall drug use compared with controls. Based on SRs valid at the time of PsA diagnosis, new-onset PsA patients had significantly higher prevalence of diabetes mellitus (women: 11% vs. 7%; men: 14% vs. 10%; all *p* < 0.001), cardiovascular diseases (women: 19% vs. 14%; men: 23% vs. 18%; all *p* < 0.001), and obstructive lung diseases (women: 12% vs. 7%; men: 8% vs. 6%; all *p* < 0.001) compared with controls. PsA patients demonstrate increased morbidity already at the time of PsA diagnosis. Screening for comorbidities should therefore be considered when joint disease is clinically apparent.

**Supplementary Information:**

The online version contains supplementary material available at 10.1007/s00296-026-06212-2.

## Introduction

Psoriatic arthritis (PsA) is a chronic inflammatory rheumatic disease (IRD) characterized by a wide spectrum of manifestations, including peripheral arthritis, dactylitis, enthesitis, axial involvement, and joint deformities [1]. Globally, the prevalence of PsA is estimated at 0.11% in adults [2], and 25–30% among patients with psoriasis (PsO) [3, 4]. Nail dystrophy, scalp lesions, perianal lesions, uveitis, elevated inflammatory markers, obesity, and low educational level have all been associated with an increased risk of PsA among patients with PsO [5–8]. In Finland, the incidence of PsA increased in the early 2000s but has since stabilized at approximately 13 per 100 000 population [9, 10].

Patients with PsA exhibit increased prevalence of comorbidities such as diabetes mellitus (DM), hypertension (HA), hyperlipidemia, anxiety, depression, and gout [11–15]. These comorbidities negatively affect quality of life and increase healthcare utilization and costs [16, 17]. Systematic screening for comorbidities in patients with IRDs is considered beneficial, and the European Alliance of Associations for Rheumatology (EULAR) has issued recommendations on screening and prevention of selected comorbidities in IRDs [18, 19].

Data on comorbidities at the earliest stages of PsA remain limited, and potential sex differences are insufficiently explored. In our previous study, we showed that the use of medications for depression and anxiety was already increased at the time of PsA diagnosis [20]. In the present study, we aimed to evaluate the prevalence of other comorbidities already at the time of PsA diagnosis.

## Materials and methods

In Finland, patients with chronic IRDs, such as PsA, may receive a 65% special reimbursement (SR) for disease-modifying anti-rheumatic drugs (DMARDs) through the Social Insurance Institution (SII). Granting an SR requires a medical certificate issued by a rheumatologist or a physician working in a rheumatology clinic. The certificate must detail the diagnostic procedures performed and the planned treatment, and each application is evaluated by an SII insurance physician. Because the SR offers substantial financial benefit, the certificate is typically issued at the same visit during which the first DMARD is prescribed, and this visit typically coincides with the clinical diagnosis of PsA.

The SII maintains a nationwide register of all individuals granted SRs, including information on sex, age, date of entitlement, and the International Classification of Disease 10th revision (ICD-10) code specifying the diagnosis for which the SR was granted. From this register, we identified all adult patients (aged ≥ 17 years) granted their first SR for DMARDs with a diagnosis of PsA (ICD-10 L40.5) between 1 January 2002 and 31 December 2014. A granted SR with PsA diagnosis was the sole inclusion criterion, and no exclusion criteria were applied. The date of the SR decision was defined as the index date (ID).

For each PsA case, three individually matched general population (GP) controls were randomly selected from Statistics Finland according to age, sex, and place of residence at the ID. Controls who had received an SR for any inflammatory arthritis before 2002 were excluded.

Data on educational attainment for PsA patients and controls were obtained from Statistics Finland, and median annual incomes (with index adjustments) were collected from SII. Education was categorized into three levels: basic (compulsory comprehensive school, grades 1–9), middle (high school, vocational school, or trade school), and high (including lower and upper tertiary levels).

SRs granted for other chronic diseases before the ID were collected for both cases and controls from the SII register (Supplementary Material 1). Data on prescription drug purchases between 1 January 2000 and 31 December 2014 were retrieved from the Drug Purchase Register (DPR), which records all reimbursed prescriptions and includes the Anatomical Therapeutic Chemical (ATC) code, quantity, and date of purchase (ATC codes listed in Supplementary Material 2). Purchases of prescription drugs were expressed as number of purchases per person-year (PY), and corresponding incidence rate ratios (IRRs) were used to compare purchases in ATC subgroups.

Additionally, we collected data on visits to specialized medical care and their primary diagnoses (ICD-10 coded) for both cases and controls between 1 January 2000 and 31 December 2014 from the Finnish Institute for Health and Welfare. ICD-10 codes used in analyses are presented in Supplementary Material 3. Risk ratios (RRs) with 95% confidence intervals (CIs) were used to compare specialized healthcare visits between incident PsA patients and their matched controls. Alcohol-related diagnoses (Supplementary Material 4) were analyzed separately. Given the incomplete coverage of primary care registry data, primary care visits were excluded.

As our objective was to assess the overall disease burden at the time of PsA diagnosis, prescription drug purchases, and primary diagnoses from specialist care visits were examined for the period from 24 months before to 3 months after the ID. The 3-month period following the ID was included to capture possible comorbidities identified at the same visit at which the PsA diagnosis was made.

The reporting of this case–control study adheres to the STROBE guidelines (EQUATOR Network), and the completed checklist is provided as Supplementary Material 5.

### Statistical methods

Data are presented as means (standard deviation, SD) or as counts (n) with percentages (%). Baseline characteristics were compared between the control and PsA groups using the chi-square test or the z test, as appropriate. Prescription drug purchases and incidences were analyzed using generalized linear models with an appropriate distribution and link function. Models were adjusted for age and sex when appropriate. Statistical analyses were performed using Stata statistical software version 18 (StataCorp, College Station, TX).

## Results

Between 2002 and 2014, we identified 6077 adult PsA patients and 18 152 matched controls. A slight majority of the cases were men (50.7%). The mean age (SD) was 48 (13) years for men and 49 (13) years for women. Women were somewhat more highly educated than men in both the PsA and control groups, and controls were slightly more highly educated than cases overall. Annual incomes did not differ between PsA patients and their same-sex controls; however, men had significantly higher incomes than women (Table 1).

**Table 1:** Study population

	Women			Men		
	Control	PsA	*P*-value	Control	PsA	*P*-value
Number	8949	2996	–	9203	3081	–
Age, mean (SD)	49 (13)	49 (13)	–	48 (13)	48 (13)	–
Incomes 1000 €, median (IQR)	31 (18, 43)	31 (18 to 41)	0.81	39 (21, 55)	40 (24, 55)	0.39
Education level N (%)			< 0.001			0.53
Basic	2001 (22)	689 (23)		2401 (26)	761 (25)	
Middle	5006 (56)	1802 (60)		5089 (55)	1816 (59)	
High	1939 (22)	505 (17)		1713 (19)	504 (16)	
Comorbidities N (%)						
DM	639 (7)	322 (11)	< 0.001	921(10)	417 (14)	< 0.001
Neurological	150 (2)	61 (2)	0.20	184 (2)	60 (2)	0.86
Psychiatric	77 (3)	72 (2)	0.051	258 (3)	60 (2)	0.010
CVDs	1251 (14)	576 (19)	< 0.001	1666 (18)	723(23)	< 0.001
OLDs	653 (7)	369 (12)	< 0.001	525 (6)	251(8)	< 0.001

Mean age (years), median annual incomes with index increase and total numbers and portions of education levels and comorbidities based on granted special reimbursements of incident psoriatic arthritis patients and their controls on the study period (defined as the 24 months preceding and the 3 months following the index date, i.e., the date on which special reimbursement for DMARDs with a PsA diagnosis became effective). Three education levels reported: Basic-level = compulsory basic comprehensive school, classes 1 to 9, middle-level = high school, vocational school and trade school, high-level = lower and upper high-levels

*SD* standard deviation, *IQR* interquartile range, *DM* diabetes mellitus, *CVDs* cardiovascular diseases, *OLDs* obstructive lung diseases

According to SRs granted for other chronic diseases, PsA patients had a higher prevalence of DM, obstructive lung diseases (OLDs) and cardiovascular diseases (CVDs) already at the time of PsA diagnosis in both sexes (*p* < 0.001). PsA men also had a higher prevalence of psychiatric disorders than their controls (*p* = 0.010). When comparing sexes, PsA women had a higher prevalence of OLDs than PsA men (12% vs. 8%), whereas PsA men had a higher prevalence of DM and CVDs than women (14% vs. 11% and 23% vs. 19%, respectively) (Table 1).

**Fig. 1:**
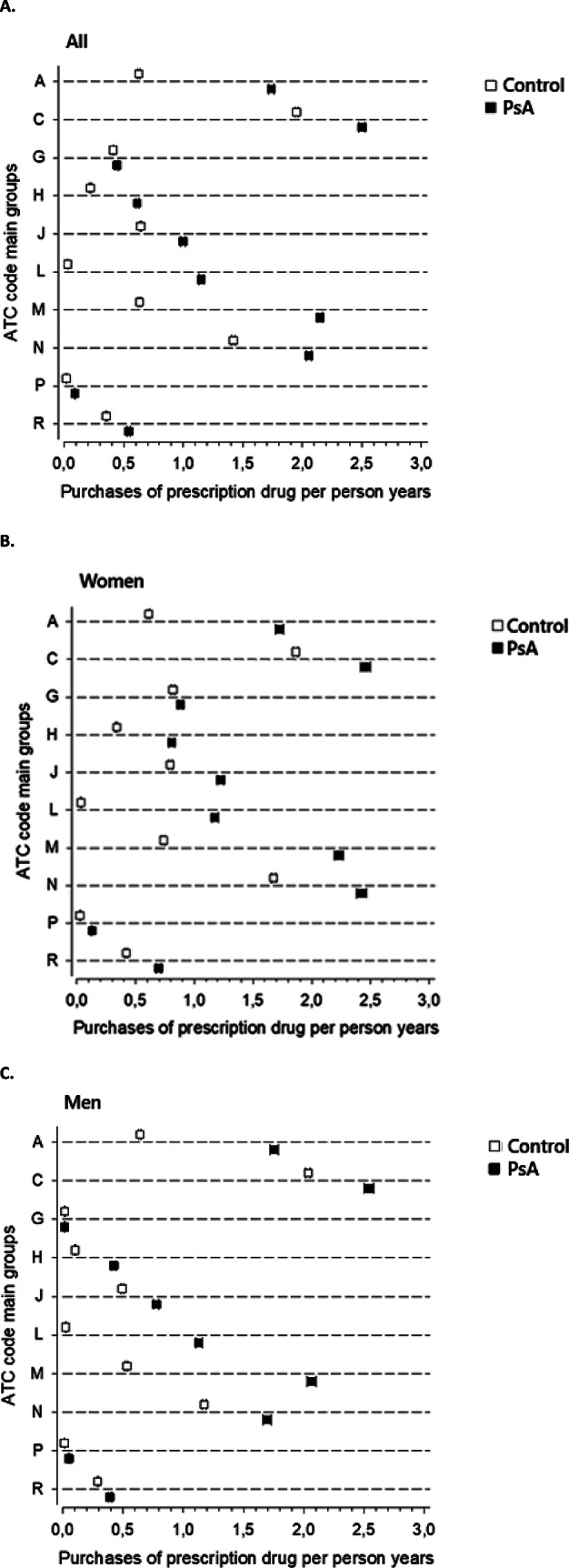
**A** Prescription drug purchases by ATC main groups. Purchases of prescription drugs per person per year, categorized by ATC main groups, for incident psoriatic arthritis patients and their matched controls during the study period (defined as the 24 months preceding and the 3 months following the index date, i.e., the date on which special reimbursement for DMARDs for PsA became effective). **B** presents corresponding data for women with PsA and their controls, and **C** for men with PsA and their controls. *A* medicines used for alimentary tract and metabolism, *C* medicines used for cardiovascular system, *G* medicines used for genito urinary system and sex hormones, *H* systemic hormonal preparations, excluding sex hormones and insulins, *J* anti-infectives for systemic use, *L* antineoplastic and immunomodulating agents, *M* medicines for musculo-skeletal system, *N* medicines for nervous system, *P* antiparasitic products, insecticides and repellents, *R* medicines used for respiratory system. *ACT code* the Anatomical Therapeutic Chemical code

Drug purchases during the study period were higher among both male and female PsA patients across all ATC main groups compared with their controls. Drugs used for CVDs represented the most frequently purchased prescription medications for both sexes during the 24 months before and 3 months after the ID. PsA women purchased more anti-infective agents, nervous system drugs, and OLD-related drugs than PsA men (Fig. 1A and B, and C).

Table 2 shows drug purchases in selected ATC subgroups per PY and the corresponding IRRs. Compared with their controls, PsA patients were more likely to purchase medications from nearly all ATC subgroups analyzed.

**Table 2:** Incidence rate ratios (IRRs) for prescription drug purchases in incident PsA patients and matched controls by ATC subgroups

ATC code	Control Mean (95% CI)	PsA Mean (95% CI)	IRR (95% CI)
A02B	0.28 (0.28 to 0.29)	0.58 (0.57 to 0.59)	**2.06 (2.00 to 2.13)**
A07A	0.00 (0.00 to 0.00)	0.00 (0.00 to 0.00)	0.60 (0.36 to 1.00)
A07E	0.03 (0.03 to 0.04)	0.71 (0.69 to 0.72)	**20.26 (19.10 to 21.49)**
A10	0.31 (0.30 to 0.31)	0.45 (0.44 to 0.47)	**1.47 (1.43 to 1.52)**
C01	0.11 (0.10 to 0.11)	0.12 (0.11 to 0.12)	1.07 (1.00 to 1.13)
C02	0.01 (0.01 to 0.01)	0.02 (0.01 to 0.02)	**1.27 (1.08 to 1.50)**
C03	0.14 (0.14 to 0.15)	0.22 (0.22 to 0.23)	**1.57 (1.49 to 1.64)**
C04	0.00 (0.00 to 0.00)	0.00 (0.00 to 0.00)	0.00 (0.00 to 0.00)
C07	0.47 (0.46 to 0.48)	0.63 (0.61 to 0.64)	**1.33 (1.30 to 1.37)**
C08	0.22 (0.22 to 0.23)	0.29 (0.28 to 0.30)	**1.28 (1.23 to 1.33)**
C09	0.58 (0.57 to 0.59)	0.77 (0.75 to 0.78)	**1.32 (1.29 to 1.36)**
C10	0.41 (0.41 to 0.42)	0.46 (0.45 to 0.48)	**1.12 (1.09 to 1.16)**
G03	0.41 (0.40 to 0.42)	0.44 (0.43 to 0.45)	**1.08 (1.04 to 1.11)**
H01	0.00 (0.00 to 0.01)	0.00 (0.00 to 0.00)	0.76 (0.53 to 1.07)
H02	0.06 (0.06 to 0.06)	0.39 (0.38 to 0.40)	**6.48 (6.16 to 6.82)**
H03	0.16 (0.15 to 0.16)	0.22 (0.21 to 0.23)	**1.43 (1.37 to 1.50)**
J01	0.58 (0.57 to 0.59)	0.90 (0.88 to 0.92)	**1.55 (1.52 to 1.59)**
J02	0.04 (0.04 to 0.05)	0.07 (0.06 to 0.07)	**1.58 (1.45 to 1.72)**
J04	0.00 (0.00 to 0.00)	0.00 (0.00 to 0.00)	**3.32 (1.35 to 8.17)**
J05	0.02 (0.02 to 0.02)	0.03 (0.03 to 0.03)	**1.39 (1.22 to 1.58)**
L01	0.01 (0.01 to 0.01)	0.19 (0.19 to 0.20)	**23.76 (21.05 to 26.82)**
L04	0.02 (0.02 to 0.02)	0.96 (0.94 to 0.98)	**46.17 (42.89 to 49.70)**
M01	0.58 (0.57 to 0.59)	2.05 (2.03 to 2.08)	**3.55 (3.49 to 3.62)**
M04	0.02 (0.02 to 0.03)	0.06 (0.06 to 0.07)	**2.60 (2.36 to 2.87)**
M05	0.03 (0.03 to 0.03)	0.03 (0.03 to 0.04)	1.08 (0.97 to 1.21)
N02	0.31 (0.31 to 0.32)	0.67 (0.66 to 0.69)	**2.17 (2.11 to 2.23)**
N03	0.15 (0.15 to 0.15)	0.21 (0.20 to 0.21)	**1.38 (1.32 to 1.45)**
N05	0.57 (0.56 to 0.57)	0.60 (0.59 to 0.62)	**1.06 (1.04 to 1.09)**
N06	0.39 (0.39 to 0.40)	0.57 (0.56 to 0.58)	**1.45 (1.41 to 1.49)**
P01	0.02 (0.02 to 0.02)	0.09 (0.08 to 0.09)	**4.66 (4.23 to 5.12)**
R03	0.35 (0.35 to 0.36)	0.54 (0.53 to 0.56)	**1.53 (1.49 to 1.58)**

Incidence rate ratios (IRRs) with 95% confidence intervals (CIs) for prescription drug purchases among incident psoriatic arthritis patients and their matched controls for the study period (24 months before and 3 months after the index date, defined as the date on which special reimbursement for DMARDs for PsA became effective), stratified by ATC code subgroups

*A02B* proton pump inhibitors, *A07A*  intestinal anti-infectives, *A07E* intestinal anti-inflammatory agents, *A10* medicines used in diabetes mellitus, *C01* medicines used in cardiac therapy, *C02* medicines used for treatment of hypertension, *C03* diuretics, *C04* peripheral vasodilators, *C07* beta block agents, *C08* calcium channel blockers, *C09* agents acting on the renin-angiotensin system, *C10* lipid modifying agents, *G03* sex hormones and modulators of the genital systems, *H01* pituitary and hypothalamic hormones and analogues, *H02* corticosteroids for systemic use, *H03* thyroid preparations, *J01* antibacterials for systemic use, *J02* antimycotics for systemic use, *J04* antimycobacterials, *J05*  antivirals for systemic use, *L01* antineoplastic agents, *L04* immunosuppressants, *M01* anti-inflammatory and antirheumatic products, *M04* antigout preparations, *M05* drugs for treatment of bone diseases, *N02*  analgesics, *N03* antiepileptics, *N05* psycholeptics, *N06* psychoanaleptics, *P01* antiprotozoal drugs, *R03* medicines used for obstructive lung diseases, *ATC code* the Anatomical Therapeutic Chemical code

Bold values indicate IRRs with CIs above 1.00

PsA patients visited specialized medical care more than three times as often as their controls during the study period of 24 months before and 3 months after the ID. Diagnoses related to the musculoskeletal system and skin diseases were the most common primary diagnoses in PsA patients. In almost every other diagnostic category, PsA patients also had more visits than controls. Only neoplasms and congenital malformations, deformations, and chromosomal abnormalities were not overrepresented among PsA patients, and neither group had visits attributed to external causes of morbidity or mortality during follow-up (Table 3). Alcohol-related primary diagnoses were rare in both groups, with no differences between cases and controls or between sexes.

**Table 3:** Risk ratios (RRs) for primary ICD-coded diagnoses from specialized medical care visits among incident PsA patients and matched controls

Primary diagnosis (ICD-code) of the visit	Control Mean (95% CI)	PsA Mean (95% CI)	RR
A00–B99	0.02 (0.02 to 0.02)	0.05 (0.04 to 0.05)	**2.35 (2.11 to 2.62)**
C00–D48	0.18 (0.17 to 0.18)	0.16 (0.15 to 0.16)	0.88 (0.83 to 0.92)
D50–D89	0.01 (0.01 to 0.01)	0.03 (0.03 to 0.03)	**2.44 (2.12 to 2.81)**
E00–E90	0.04 (0.03 to 0.04)	0.05 (0.05 to 0.06)	**1.49 (1.35 to 1.63)**
F00–F99	0.21 (0.21 to 0.22)	0.25 (0.24 to 0.26)	**1.19 (1.14 to 1.24)**
G00–G99	0.07 (0.07 to 0.07)	0.12 (0.11 to 0.13)	**1.66 (1.56 to 1.77)**
H00–H59	0.08 (0.07 to 0.08)	0.12 (0.11 to 0.12)	**1.53 (1.43 to 1.63)**
I00–I99	0.11 (0.10 to 0.11)	0.11 (0.11 to 0.12)	**1.08 (1.01 to 1.14)**
J00–J99	0.05 (0.05 to 0.06)	0.09 (0.09 to 0.10)	**1.67 (1.55 to 1.80)**
K00–K93	0.08 (0.08 to 0.09)	0.14 (0.13 to 0.14)	**1.64 (1.55 to 1.75)**
L00–L99	0.04 (0.04 to 0.05)	1.35 (1.32 to 1.37)	**30.65 (29.11 to 32.27)**
M00–M99	0.14 (0.13 to 0.14)	1.51 (1.49 to 1.53)	**11.19 (10.84 to 11.55)**
N00–N99	0.08 (0.08 to 0.08)	0.08 (0.08 to 0.09)	1.05 (0.98 to 1.13)
Q00–Q99	0.01 (0.01 to 0.01)	0.00 (0.00 to 0.01)	0.49 (0.37 to 0.64)
R00–R99	0.09 (0.09 to 0.09)	0.15 (0.14 to 0.15)	**1.65 (1.56 to 1.75)**
S00–T98	0.09 (0.09 to 0.10)	0.11 (0.10 to 0.12)	**1.16 (1.09 to 1.24)**
V01–Y98	0.00 (0.00 to 0.00)	0.00 (0.00 to 0.00)	0.00 (0.00 to 0.00)
Z00–ZZB	0.12 (0.11 to 0.12)	0.19 (0.18 to 0.20)	**1.61 (1.53 to 1.70)**
All together	1.46 (1.45 to 1.47)	4.54 (4.50 to 4.58)	3.11 **(3.08 to 3.15)**

Risk ratios (RRs) with 95% confidence intervals (CIs) for primary ICD-coded diagnoses from specialized medical care visits among incident psoriatic arthritis patients and their matched controls for the study period (24 months before and 3 months after the index date, defined as the date on which special reimbursement for DMARDs for PsA became effective), adjusted for sex and age

*ICD-code* the International Classification of Disease 10th revision code, *A00–B99* certain infectious and parasitic diseases, *C00-D48* neoplasms, *D50–D89* diseases of the blood and blood-forming organs and certain disorders involving the immune mechanism, *E00–E90* endocrine, nutritional and metabolic diseases; *F00–F99* mental and behavioral disorders, *G00–G99* diseases of the nervous, *H00–H59* diseases of the eye and adnexa, *I00–I99* diseases of the circulatory system, *J00–J99* diseases of the respiratory system, *K00–K93* diseases of the digestive system, *L00–L99* diseases of the skin and subcutaneous tissue, *M00–M99* diseases of the musculoskeletal system and connective tissue, *N00–N99* diseases of the genitourinary system, *Q00–Q99* congenital malformations, deformations and chromosomal abnormalities, *R00–R99* symptoms, signs and abnormal clinical and laboratory findings, not elsewhere classified, *S00–T98* injury, poisoning and certain other consequences of external causes, *V01–Y98* external causes of morbidity and mortality, *Z00–ZZB* factors influencing health status and contact with health services

Bold values indicate RRs with CIs above 1.00

## Discussion

In this study, we demonstrated that PsA patients experience an increased disease burden already at the time of the diagnosis. This was reflected in higher drug consumption, more frequent specialized medical care visits, and more SRs granted for DM, CVDs, and OLDs. In addition, men with PsA were more frequently granted SRs for psychiatric disorders than their controls.

Previous studies on PsA comorbidities have primarily focused on established joint disease. We specifically aimed to investigate the burden of comorbidities in patients with incident PsA from a rheumatological perspective, and to assess potential sex-specific differences.

The prevalence of hypertension, hyperlipidemia, type 2 DM, and cardiovascular events has repeatedly been shown to be higher in PsA patients than in the general population (GP) or in PsO patients without arthritis [11, 12, 21–24]. A UK database study found that PsA patients not treated with DMARDs had a greater risk of major adverse cardiovascular events (MACE) compared with those receiving DMARDs [25]. Dysregulated lipid metabolism may contribute to both PsO and PsA pathogenesis and may help explain their association with CVDs [26].

Our results also suggest an association between PsA and asthma as well as COPD, although behavioral risk factors such as smoking were not available. Asthma is generally more common and more severe among women [27], and women develop COPD with less exposure and experience a greater symptom burden than men [28]. Our findings are consistent with these patterns: PsA women had more OLDs and purchased more OLD-related drugs than PsA men. Importantly, both sexes had significantly more OLDs than their matched controls. PsA patients also attended specialized medical care more frequently for respiratory diagnoses (ICD-10 J00–J99). Several studies have suggested links between allergy-related diseases and autoimmune disorders, as well as associations between asthma and PsO [29–31], although results remain inconsistent [32]. Associations between PsO/PsA and COPD have also been reported [16, 33]. One potential mechanistic explanation is the shared involvement of phosphodiesterase 4 (PDE-4); apremilast is used to treat PsO and PsA, whereas roflumilast is used for COPD [34].

Consistent with our previous findings [20], purchases of drugs used for depression and anxiety were already increased before PsA diagnosis. PsA men were also granted more SRs for psychiatric conditions than their controls, suggesting a higher burden of severe psychiatric disorders.

We also observed increased purchases of antibiotics among new-onset PsA patients, as well as more medical visits for infectious disease diagnoses (A00–B99). This may indicate a higher rate of infections preceding or accompanying PsA onset. While infections have been studied as possible triggers for PsO [35], data regarding infections and incident PsA remain scarce.

Patients with new-onset PsA more frequently purchased thyroid medications than their controls, suggesting a possible association between PsA development and autoimmune thyroid disorders. Altered thyroid hormone levels have been implicated in PsO pathogenesis [36], and a large cross-sectional study found an association between PsO and Hashimoto’s thyroiditis [37].

Although PsA has been proposed as an independent risk factor for decreased bone density [38], we did not observe higher use of osteoporosis medications among PsA patients than among controls, despite their higher glucocorticoid use.

Alcohol consumption has previously been associated with both increased risk of PsA and lower prevalence of peripheral arthritis [39, 40]. In our data, alcohol-related diagnoses requiring specialist care were rare in both groups, with no differences between cases and controls.

As expected, PsA patients purchased drugs in ATC groups H02, L01, L04, and M01 [including corticosteroids for systemic use, methotrexate (MTX), biological DMARDs (bDMARDs), and non-steroidal anti-inflammatory drugs (NSAIDs)] more frequently than controls. PsO preceding PsA and joint symptoms related PsA are likely to contribute to these findings. The use of sulfasalazine and hydroxychloroquine in the treatment of joint symptoms likely explains the differences observed in intestinal anti-inflammatory agents (A07E) and antiprotozoal drugs (P01). Increased glucocorticoid and NSAID use likely contributed to greater proton pump inhibitor (A02B) purchases among PsA patients. The higher use of analgesics (N02) in new-onset PsA patients aligns with our previous study, in which initiation of DMARDs did not significantly reduce opioid use [20].

Socioeconomic factors, such as lower income and lower educational level, are known to increase the incidence of DM and CVDs [41, 42]. In a Danish study using the Charlson Comorbidity Index (CCI), women with PsA had higher CCI scores [43]. In our study, no income differences were observed between PsA patients and same-sex controls. However, women in both groups had lower incomes than men, and higher educational attainment—particularly among women—was more common among controls than cases. Although comorbidity burden was similar between sexes, women may experience its consequences more heavily due to socioeconomic disadvantage.

Our findings of increased healthcare use among new-onset PsA patients are consistent with a U.S. claims-based study including prevalent PsA patients [16]. Another U.S. study, focusing also on patients with established PsA, showed elevated healthcare costs, hospitalization rates, emergency department visits, and outpatient service utilization among patients with established PsA and concomitant moderate-to-severe PsO compared with controls [17]. Kaine et al. reported that patients with newly diagnosed PsA have a markedly increased comorbidity burden compared with matched controls, including cardiovascular and autoimmune diseases [44]. While comorbidity profiles in PsA have been extensively characterized in these U.S. claims-based studies, including among newly diagnosed patients, analyses stratified by sex remain limited. In a multicenter study conducted in India, the prevalence of comorbidities in PsA was strongly associated with age, with older patients (≥ 40 years) showing a substantially higher burden [45]. In our study, the mean age of patients with PsA was 49 years in women and 48 years in men. In a cross-sectional study from Poland comparing comorbidities across IRDs, obesity was more prevalent among patients with PsA than among those with rheumatoid arthritis (RA) and axial spondyloarthritis (axSpA) [46]. In a study of newly diagnosed, treatment-naïve PsA patients, cardiovascular risk factors were already more prevalent compared with controls at baseline and remained unchanged during one year of follow-up despite improvements in disease activity [47].

As expected, visits related to skin (L00–L99) and musculoskeletal (M00–M99) diagnoses were most common among PsA patients, likely reflecting care related to PsO and early PsA manifestations. Thus, our results probably mirror the comorbidity profile of patients with long-standing or severe PsO.

The main strengths of our register-based study include the use of comprehensive, high-quality national registers, enabling identification of virtually all Finnish PsA patients initiating DMARD therapy. Reliability of PsA diagnoses is high, as SR certificates must be authored by a rheumatologist or issued at a rheumatology clinic. The use of three complementary methods to detect comorbidities—SRs, drug purchases, and specialist care diagnoses—is also a major strength of the study and enables the assessment of comorbidities from multiple perspectives.

The main limitation is the lack of clinical and behavioral data. For example, obesity, smoking, and alcohol consumption—well-established risk factors for many comorbidities and for PsA—could not be analyzed. Based on the available registry data, the date on which SR for DMARD therapy is granted represents the most accurate proxy for the timing of PsA diagnosis. However, in patients with milder symptoms, whose disease is managed with local glucocorticoid therapy and NSAIDs, SR for DMARDs is not necessarily applied for at the time of diagnosis. These patients may be underrepresented in our cohort or have a longer diagnostic delay. In turn, perhaps reflecting the challenges in diagnosing PsA, in our previous study, up to 31% of patients granted SR for DMARDs due to PsA between 2010 and 2014 had already purchased MTX during the year preceding the SR decision [20]. Intravenous treatments administered in hospitals were unavailable, but since conventional synthetic DMARD (csDMARD) trials are required before bDMARD initiation, few patients are likely to be missing [20]. We analyzed only specialist care visits, potentially focusing on more severe comorbidities, as primary care visits were excluded. In contrast, analyses of medication purchases also captured conditions treated in primary care. Once referred to specialist healthcare services, patients are more likely to undergo further consultations and receive additional diagnoses, which may contribute to an increased comorbidity burden among incident PsA patients.

This epidemiological study provides a comprehensive assessment of the comorbidity burden among patients with incident PsA within the Finnish healthcare system and based on nationwide register data; consequently, the findings may not be directly generalisable to other healthcare settings or populations.

In summary, PsA patients already exhibit an increased comorbidity burden at the time of joint disease diagnosis. Because PsO precedes PsA in the majority of cases [48], our findings support early and proactive screening and prevention of comorbidities among patients with PsO. Early identification and management of comorbidities may help reduce overall disease burden and improve long-term outcomes in PsA.

## Supplementary Information

Below is the link to the electronic supplementary material.

Supplementary Material 1

Supplementary Material 2

Supplementary Material 3

Supplementary Material 4

Supplementary Material 5

## Data Availability

Due to ethical and legal considerations, the data supporting this study are not publicly available.

## Declarations

### Disclaimer

Authors certify that this work is original, has not been published previously, or generated by AI. Microsoft Copilot was used for language editing. Authors reviewed and revised the text and take full responsibility for the final manuscript.

## References

[1] Kishimoto M, Deshpande GA, Fukuoka K, Kawakami T, Ikegaya N, Kawashima S et al (2021) Clinical features of psoriatic arthritis. Best Pract Res Clin Rheumatol 35(2):101670. 10.1016/j.berh.2021.101670 33744078 10.1016/j.berh.2021.101670

[2] Lembke S, Macfarlane GJ, Jones GT (2024) The worldwide prevalence of psoriatic arthritis—a systematic review and meta-analysis. Rheumatology 63(12):3211–3220. 10.1093/rheumatology/keae198 38530786 10.1093/rheumatology/keae198 PMC11637478

[3] Loo YP, Loo CH, Lim AL, Wong CK, Ali NBM, Khor YH et al (2023) Prevalence and risk factors associated with psoriatic arthritis among patients with psoriasis. Int J Rheum Dis 26(9):1788–1798. 10.1111/1756-185X.14833 37485806 10.1111/1756-185X.14833

[4] Alinaghi F, Calov M, Kristensen LE, Gladman DD, Coates LC, Jullien D et al (2019) Prevalence of psoriatic arthritis in patients with psoriasis: a systematic review and meta-analysis of observational and clinical studies. J Am Acad Dermatol 80(1):251–265e19. 10.1016/j.jaad.2018.06.027 29928910 10.1016/j.jaad.2018.06.027

[5] Kılıç G, Kılıç E, Tekeoğlu İ, Sargın B, Cengiz G, Balta NC (2025) Factors influencing the transition time from psoriasis to psoriatic arthritis: a real-world multicenter analysis. Rheumatol Int 45(9):225. 10.1007/s00296-025-05984-3 40924166 10.1007/s00296-025-05984-3

[6] Yao A, Wang L, Qi F, Li J, Meng J, Jiang T et al (2024) Risk factors and early detection of joint damage in patients with psoriasis: a case–control study. Int J Dermatol 63(12):1728–1734. 10.1111/ijd.17212 10.1111/ijd.17212 38682296

[7] Eder L, Haddad A, Rosen CF, Lee K, Chandran V, Cook R et al (2016) The incidence and risk factors for psoriatic arthritis in patients with psoriasis: a prospective cohort study. Arthritis Rheumatol 68(4):915–923. 10.1002/art.39494 26555117 10.1002/art.39494

[8] Jon Love T, Zhu Y, Zhang Y, Wall-Burns L, Ogdie A, Gelfand JM et al (2012) Obesity and the risk of psoriatic arthritis: a population-based study. Ann Rheum Dis 71(8):1273–1277. 10.1136/annrheumdis-2012-201299 22586165 10.1136/annrheumdis-2012-201299 PMC3645859

[9] Muilu P, Rantalaiho V, Kautiainen H, Virta LJ, Eriksson JG, Puolakka K (2019) Increasing incidence and shifting profile of idiopathic inflammatory rheumatic diseases in adults during this millennium. Clin Rheumatol 38(2):555–562. 10.1007/s10067-018-4310-0 30259249 10.1007/s10067-018-4310-0

[10] Heimola L, Peltomaa R, Laine A, Kautiainen H, Puolakka K, Rantalaiho V (2025) Evolution of various inflammatory arthritis incidences during 2015–2020: a nationwide population-based register study in Finland. Jt Bone Spine 92(4):105886. 10.1016/j.jbspin.2025.105886 10.1016/j.jbspin.2025.105886 40090615

[11] Shah K, Paris M, Mellars L, Changolkar A, Mease PJ (2017) Real-world burden of comorbidities in US patients with psoriatic arthritis. RMD Open 3(2):e000588. 10.1136/rmdopen-2017-000588 29435363 10.1136/rmdopen-2017-000588 PMC5761305

[12] Dubreuil M, Rho YH, Man A, Zhu Y, Zhang Y, Love TJ et al (2014) Diabetes incidence in psoriatic arthritis, psoriasis and rheumatoid arthritis: a UK population-based cohort study. Rheumatology 53(2):346–352. 10.1093/rheumatology/ket343 24185762 10.1093/rheumatology/ket343 PMC3894671

[13] Edson-Heredia E, Zhu B, Lefevre C, Wang M, Barrett A, Bushe CJ et al (2015) Prevalence and incidence rates of cardiovascular, autoimmune, and other diseases in patients with psoriatic or psoriatic arthritis: a retrospective study using clinical practice research datalink. Acad Dermatol Venereol 29(5):955–963. 10.1111/jdv.12742 10.1111/jdv.12742 25352213

[14] Zhao SS, Miller N, Harrison N, Duffield SJ, Dey M, Goodson NJ (2020) Systematic review of mental health comorbidities in psoriatic arthritis. Clin Rheumatol 39(1):217–225. 10.1007/s10067-019-04734-8 31486931 10.1007/s10067-019-04734-8

[15] Merola JF, Wu S, Han J, Choi HK, Qureshi AA (2015) Psoriasis, psoriatic arthritis and risk of gout in US men and women. Ann Rheum Dis 74(8):1495–1500. 10.1136/annrheumdis-2014-205212 24651620 10.1136/annrheumdis-2014-205212 PMC4224633

[16] Merola JF, Herrera V, Palmer JB (2018) Direct healthcare costs and comorbidity burden among patients with psoriatic arthritis in the USA. Clin Rheumatol 37(10):2751–2761. 10.1007/s10067-018-4187-y 30051284 10.1007/s10067-018-4187-y

[17] Feldman SR, Zhao Y, Shi L, Tran MH, Lu J (2015) Economic and comorbidity burden among moderate-to‐severe psoriasis patients with comorbid psoriatic arthritis. Arthritis Care Res 67(5):708–717. 10.1002/acr.22492 10.1002/acr.22492 PMC5029589 25303478

[18] Immediato Daien C, Georgescu V, Decarriere G, Mercier G, Morel J (2024) Systematic screening for multimorbidities in patients with inflammatory rheumatic diseases enhanced preventive medication use and reduced hospitalisations: an exposed-non-exposed study. RMD Open 10(4):e004490. 10.1136/rmdopen-2024-004490 39357925 10.1136/rmdopen-2024-004490 PMC11448191

[19] Baillet A, Gossec L, Carmona L, Wit MD, Van Eijk-Hustings Y, Bertheussen H et al (2016) Points to consider for reporting, screening for and preventing selected comorbidities in chronic inflammatory rheumatic diseases in daily practice: a EULAR initiative. Ann Rheum Dis 75(6):965–973. 10.1136/annrheumdis-2016-209233 26984008 10.1136/annrheumdis-2016-209233

[20] Laine A, Muilu P, Kautiainen H, Puolakka K, Rantalaiho V (2025) Pain management, prolonged opioid use, initiated anti-rheumatic treatment and psychiatric morbidity in new-onset psoriatic arthritis. Rheumatol Adv Pract 9(2):rkaf039. 10.1093/rap/rkaf039 40352325 10.1093/rap/rkaf039 PMC12064172

[21] Gladman DD, Ang M, Su L, Tom BDM, Schentag CT, Farewell VT (2009) Cardiovascular morbidity in psoriatic arthritis. Ann Rheum Dis 68(7):1131–1135. 10.1136/ard.2008.094839 18697777 10.1136/ard.2008.094839

[22] Dal Bello G, Gisondi P, Idolazzi L, Girolomoni G (2020) Psoriatic arthritis and diabetes mellitus: a narrative review. Rheumatol Ther 7(2):271–285. 10.1007/s40744-020-00206-7 32306243 10.1007/s40744-020-00206-7 PMC7211212

[23] Husted JA, Thavaneswaran A, Chandran V, Eder L, Rosen CF, Cook RJ et al (2011) Cardiovascular and other comorbidities in patients with psoriatic arthritis: a comparison with patients with psoriasis. Arthritis Care Res 63(12):1729–1735. 10.1002/2Facr.20627 10.1002/acr.20627 21905258

[24] Charlton R, Green A, Shaddick G, Snowball J, Nightingale A, Tillett W et al (2019) Risk of type 2 diabetes and cardiovascular disease in an incident cohort of people with psoriatic arthritis: a population-based cohort study. Rheumatology 58(1):144–148. 10.1093/rheumatology/key286 30202906 10.1093/rheumatology/key286

[25] Ogdie A, Yu Y, Haynes K, Love TJ, Maliha S, Jiang Y et al (2015) Risk of major cardiovascular events in patients with psoriatic arthritis, psoriasis and rheumatoid arthritis: a population-based cohort study. Ann Rheum Dis 74(2):326–332. 10.1136/annrheumdis-2014-205675 25351522 10.1136/annrheumdis-2014-205675 PMC4341911

[26] Gao Y, Yi X, Ding Y (2017) Combined transcriptomic analysis revealed AKR1B10 played an important role in psoriasis through the dysregulated lipid pathway and overproliferation of keratinocyte. Biomed Res Int 2017:1–10. 10.1155/2017/8717369 10.1155/2017/8717369 PMC5674492 29204449

[27] Zein JG, Erzurum SC (2015) Asthma is Different in Women. Curr Allergy Asthma Rep 15(6):28. 10.1007/s11882-015-0528-y 26141573 10.1007/s11882-015-0528-y PMC4572514

[28] Barnes PJ (2016) Sex differences in chronic obstructive pulmonary disease mechanisms. Am J Respir Crit Care Med 193(8):813–814. 10.1164/rccm.201512-2379ED 27082528 10.1164/rccm.201512-2379ED

[29] Krishna MT, Subramanian A, Adderley NJ, Zemedikun DT, Gkoutos GV, Nirantharakumar K (2019) Allergic diseases and long-term risk of autoimmune disorders: longitudinal cohort study and cluster analysis. Eur Respir J 54(5):1900476. 10.1183/13993003.00476-2019 31413164 10.1183/13993003.00476-2019

[30] Joel MZ, Fan R, Damsky W, Cohen JM (2023) Psoriasis associated with asthma and allergic rhinitis: a US-based cross-sectional study using the All of US Research Program. Arch Dermatol Res 315(6):1823–1826. 10.1007/s00403-023-02539-z 36707438 10.1007/s00403-023-02539-z

[31] Han JH, Bang CH, Han K, Ryu JY, Lee JY, Park YM et al (2021) The risk of psoriasis in patients with allergic diseases: a nationwide population-based cohort study. Allergy Asthma Immunol Res 13(4):638. 10.4168/aair.2021.13.4.638 34212549 10.4168/aair.2021.13.4.638 PMC8255348

[32] Zhao D, Wu S, Wang Y, Zheng H, Zhu M (2024) Association between allergic diseases and both psoriasis and psoriatic arthritis: a bidirectional 2-sample Mendelian randomization study. Arch Dermatol Res 316(5):181. 10.1007/s00403-024-02978-2 38762688 10.1007/s00403-024-02978-2

[33] Chiang Y-Y, Lin H‐W (2012) Association between psoriasis and chronic obstructive pulmonary disease: a population‐based study in Taiwan. Acad Dermatol Venereol 26(1):59–65. 10.1111/j.1468-3083.2011.04009.x 10.1111/j.1468-3083.2011.04009.x 21388457

[34] Fan T et al (2024) PDE4 inhibitors: potential protective effects in inflammation and vascular diseases. Front Pharmacol 15:1407871. 10.3389/fphar.2024.1407871 38915460 10.3389/fphar.2024.1407871 PMC11194378

[35] Liu S, He M, Jiang J, Duan X, Chai B, Zhang J et al (2024) Triggers for the onset and recurrence of psoriasis: a review and update. Cell Commun Signal 22(1):108. 10.1186/s12964-023-01381-0 38347543 10.1186/s12964-023-01381-0 PMC10860266

[36] Arican O, Bilgic K, Koc K (2004) The effect of thyroid hormones in psoriasis vulgaris. Indian J Dermatol Venereol Leprol 70(6):353–356 17642662

[37] Kiguradze T, Bruins FM, Guido N, Bhattacharya T, Rademaker A, Florek AG et al (2017) Evidence for the association of Hashimoto’s thyroiditis with psoriasis: a cross-sectional retrospective study. Int J Dermatol 56(5):553–556. 10.1111/ijd.13459 10.1111/ijd.13459 PMC5383506 28217937

[38] Wang Y, Song ZB, Deng XR, Zhang XH, Zhang ZL (2021) Risk factors associated with osteoporosis and fracture in psoriatic arthritis. Chin Med J (Engl) 134(21):2564–2572. 10.1097/CM9.0000000000001810 34670248 10.1097/CM9.0000000000001810 PMC8577663

[39] Wu S, Cho E, Li WQ, Han J, Qureshi AA (2015) Alcohol intake and risk of incident psoriatic arthritis in women. J Rheumatol 42(5):835–840. 10.3899/jrheum.140808 25834201 10.3899/jrheum.140808 PMC4600066

[40] Ladehesa-Pineda ML, Ortega-Castro R, Puche-Larrubia MÁ, Granados REM, Dougados M, Collantes-Estévez E et al (2023) Smoking and alcohol consumption are associated with peripheral musculoskeletal involvement in patients with spondyloarthritis (including psoriatic arthritis). Results from the ASAS-PerSpA study. Semin Arthritis Rheum 58:152146. 10.1016/j.semarthrit.2022.152146 36516482 10.1016/j.semarthrit.2022.152146

[41] Chen Y, Zhou X, Bullard KM, Zhang P, Imperatore G, Rolka DB (2023) Income-related inequalities in diagnosed diabetes prevalence among US adults, 2001-2018. PLoS One 18(4):e0283450. 10.1371/journal.pone.0283450 10.1371/journal.pone.0283450 PMC10101461 37053158

[42] Hahad O, Gilan DA, Chalabi J, Al-Kindi S, Schuster AK, Wicke F et al (2024) Cumulative social disadvantage and cardiovascular disease burden and mortality. Eur J Prev Cardiol 31(1):40–48. 10.1093/eurjpc/zwad264 37721449 10.1093/eurjpc/zwad264

[43] Ballegaard C, Højgaard P, Dreyer L, Cordtz R, Jørgensen TS, Skougaard M et al (2018) Impact of comorbidities on tumor necrosis factor inhibitor therapy in psoriatic arthritis: a population-based cohort study. Arthritis Care Res 70(4):592–599. 10.1002/2Facr.23333 10.1002/acr.23333 28772007

[44] Kaine J, Song X, Kim G, Hur P, Palmer JB (2019) Higher incidence rates of comorbidities in patients with psoriatic arthritis compared with the general population using U.S. Administrative claims data. JMCP 25(1):122–132. 10.18553/jmcp.2018.17421 29694270 10.18553/jmcp.2018.17421 PMC10397587

[45] Chandrashekara S, Shenoy P, Kumar U, Pandya S, Ghosh A, Khare A (2025) Increase in comorbidities with age among patients with psoriatic arthritis: a multicenter observational study. Rheumatol Int 45(1):23. 10.1007/s00296-024-05760-9 39786433 10.1007/s00296-024-05760-9

[46] Guła Z, Łosińska K, Kuszmiersz P, Strach M, Nowakowski J, Biedroń G (2024) ym. A comparison of comorbidities and their risk factors prevalence across rheumatoid arthritis, psoriatic arthritis and axial spondyloarthritis with focus on cardiovascular diseases: data from a single center real-world cohort. Rheumatol Int 44(12):2817–2828. 10.1007/s00296-024-05740-z 39527279 10.1007/s00296-024-05740-z PMC11618134

[47] Ishchenko A, Pazmino S, Neerinckx B, Lories R, De Vlam K (2024) Comorbidities in early psoriatic arthritis: data from the metabolic disturbances in psoriatic arthritis cohort study. Arthritis Care Res 76(2):231–240. 10.1002/acr.25230 10.1002/acr.25230 37667975

[48] Li J, Xiao J, Xie X, Deng S, Zhou G, Wang R et al (2024) Individual joints involvement pattern in psoriatic arthritis: a cross-sectional study in China. J Dermatol 51(12):1607–1614. 10.1111/1346-8138.17369 38995193 10.1111/1346-8138.17369

