# Supplementary material for: Psoriatic arthritis patients have increased morbidity already at the time of diagnosis: a case–control study
Source: Rheumatol Int. 2026 Jul 6;46(7):189. doi: 10.1007/s00296-026-06212-2 (PMC13337795; doi:10.1007/s00296-026-06212-2)
Supplement: Supplementary file 1 — Supplementary Material 1 [file 296_2026_6212_MOESM1_ESM.docx]

**Supplementary Material 1:**

The list of the other chronic diseases granted a special reimbursement before the index date (ID: the date when special reimbursement for anti-rheumatic drugs came into effect).

- Insulin-dependent DM (ICD-10 codes E10-E14, E89.1)
- Specific neurological diseases such as multiple sclerosis, Parkinson’s disease and epilepsy (G35, G20, G23, G24.1, G24.8, G90.3, C71, G40, G41)
- Severe psychiatric diseases such as schizophrenia, bipolar disorder, mania and depression with psychotic features (F01, F03, F06.0-F06.3, F20-F25, F28, F29, F30.1, F30.1, F30.2, F31, F32.3, F33.3, F84, G10, G20,G30.0, G30.1, G30.8, G30.9, G31.0, G35, G40.9)
- Cardiovascular diseases such as heart insufficiency, difficult chronic arterial hypertension, certain arrhythmical conditions e.g. atrial fibrillation, and chronic ischemic heart disease (I10-I13, I15, I20-I22, I24.0, I25, I27.0, I27.2, I47-I49, I50, I97.1, P29.0)
- Asthma and chronic obstructive pulmonary disease (E84.0, J41-J45, P27.1)
