# Supplementary material for: Psoriatic arthritis patients have increased morbidity already at the time of diagnosis: a case–control study
Source: Rheumatol Int. 2026 Jul 6;46(7):189. doi: 10.1007/s00296-026-06212-2 (PMC13337795; doi:10.1007/s00296-026-06212-2)
Supplement: Supplementary file 2 — Supplementary Material 2 [file 296_2026_6212_MOESM2_ESM.docx]

**Supplementary Material 2:**

The Anatomical Therapeutic Chemical (ATC) classification system main codes used to obtain the data:

- A (alimentary tract and metabolism)
- C (cardiovascular system)
- G (genito urinary system and sex hormones)
- H (systemic hormonal preparations, excluding sex hormones and insulins)
- J (anti-infectives for systemic use)
- L (antineoplastic and immunomodulating agents)
- M (musculoskeletal system)
- N (nervous system)
- P (antiparasitic products, insecticides and repellents)
- R (respiratory system).

The ATC classification system subgroup codes used to obtain the data:

- A02B (proton pump inhibitors)
- A07A (intestinal anti-infectives)
- A07E (intestinal anti-inflammatory agents)
- A10 (medicines used in DM)
- C01 (medicines used in cardiac therapy)
- C02 (medicines used for treatment of hypertension)
- C03 (diuretics)
- C04 (peripheral vasodilators)
- C07 (beta block agents)
- C08 (calcium channel blockers)
- C09 (agents acting on the renin-angiotensin system)
- C10 (lipid modifying agents)
- G03 (sex hormones and modulators of the genital systems)
- H01 (pituitary and hypothalamic hormones and analogues)
- H02 (corticosteroids for systemic use)
- H03 (thyroid preparations)
- J01 (antibacterials for systemic use)
- J02 (antimycotics for systemic use)
- J04 (antimycobacterials)
- J05 (antivirals for systemic use)
- L01 (antineoplastic agents)
- L04 (immunosuppressants)
- M01 (anti-inflammatory and antirheumatic products)
- M04 (antigout preparations)
- M05 (medicines for treatment of bone diseases)
- N02 (analgesics), N03 (antiepileptics)
- N05 (psycholeptics)
- N06 (psychoanaleptics)
- P01 (antiprotozoal drugs)
- R03 (medicines used for OLDs)
