# Supplementary material for: Psoriatic arthritis patients have increased morbidity already at the time of diagnosis: a case–control study
Source: Rheumatol Int. 2026 Jul 6;46(7):189. doi: 10.1007/s00296-026-06212-2 (PMC13337795; doi:10.1007/s00296-026-06212-2)
Supplement: Supplementary file 3 — Supplementary Material 3 [file 296_2026_6212_MOESM3_ESM.docx]

**Supplementary Material 3:**

The ICD-10 codes of the primary diagnoses used to obtain the data from the visits to specialized medical care:

- A00–B99: Certain infectious and parasitic diseases
- C00-D48: Neoplasms
- D50–D89: Diseases of the blood and blood-forming organs and certain disorders involving the immune mechanism
- E00–E90: Endocrine, nutritional and metabolic diseases
- F00–F99: Mental and behavioral disorders
- G00–G99: Diseases of the nervous
- H00–H59: Diseases of the eye and adnexa
- I00–I99: Diseases of the circulatory system
- J00–J99: Diseases of the respiratory system
- K00–K93: Diseases of the digestive system
- L00–L99: Diseases of the skin and subcutaneous tissue
- M00–M99: Diseases of the musculoskeletal system and connective tissue
- N00–N99: Diseases of the genitourinary system
- Q00–Q99: Congenital malformations, deformations and chromosomal abnormalities
- R00–R99: Symptoms, signs and abnormal clinical and laboratory findings, not elsewhere classified
- S00–T98: Injury, poisoning and certain other consequences of external causes
- V01–Y98: External causes of morbidity and mortality
- Z00–ZZB: Factors influencing health status and contact with health services

ICD-10 code class O00-O99 (pregnancy, childbirth and the puerperium) was excluded concerning women only and class P00-P96 (certain conditions originating in the perinatal period) was excluded concerning only newborns.
