# Supplementary material for: Psoriatic arthritis patients have increased morbidity already at the time of diagnosis: a case–control study
Source: Rheumatol Int. 2026 Jul 6;46(7):189. doi: 10.1007/s00296-026-06212-2 (PMC13337795; doi:10.1007/s00296-026-06212-2)
Supplement: Supplementary file 4 — Supplementary Material 4 [file 296_2026_6212_MOESM4_ESM.docx]

**Supplementary Material 4:**

The alcohol-related ICD-10 codes used to obtain the data:

- F10: Alcohol related disorders
- G31.2: Degeneration of nervous system due to alcohol
- G40.51: Epileptic seizure related to alcohol
- G62.1: Alcohol polyneuropathy
- G72.1: Alcohol myopathy
- I42.6: Alcohol cardiomyopathy
- K29.2: Alcohol gastritis
- K85.2: Alcohol-induced acute pancreatitis
- K86.0: Alcohol-induces chronic pancreatitis
- K70: Alcohol liver disease
- O35.4: Maternal care for damage to fetus from alcohol
- P04.3: Fetus and newborn affected by maternal use of alcohol
- Q86.0: Fetal alcohol syndrome
- X45: Accidental poisoning by and exposure to alcohol
